# Demographic and Clinical Characteristics Influencing Ecological Momentary Assessment Compliance in Individuals With Bipolar Disorder: Observational Study

**DOI:** 10.2196/74223

**Published:** 2026-01-07

**Authors:** Linru Yin, Minghui Li, Jinhao Li, Xiaofei Hou, Jianan Song, Xinyan Li, Yue Tong, Xinyue Zhou, Lidan Yuan, Huifang Yin, Guangming Xu

**Affiliations:** 1 Mental Health Center of Tianjin Medical University Tianjin Anding Hospital Tianjin China; 2 Xi’an Children’s Hospital Xi’an, Shaanxi China; 3 Peking University HuiLongGuan Clinical Medical School Beijing HuiLongGuan Hospital Beijing China

**Keywords:** ecological momentary assessment, bipolar disorder, patient compliance, self assessment, adherence

## Abstract

**Background:**

Bipolar disorder requires immediate and frequent daily symptom monitoring due to its extreme mood fluctuations. Ecological momentary assessment (EMA) technology uses high-frequency data collection to achieve ecologically valid capture of patient symptoms. Investigating EMA compliance among Chinese patients with bipolar disorder and its influencing factors is essential for developing more feasible daily symptom monitoring protocols.

**Objective:**

This study aimed to investigate the 14-day compliance rate of EMA among Chinese individuals with bipolar disorder and to examine the demographic and clinical characteristics associated with that compliance.

**Methods:**

A total of 100 adults (63 female individuals) with bipolar disorder across mood states (depressive episode, n=29, 29%; hypomanic or manic episode, n=17, 17%; euthymic state, n=54, 54%) completed self-monitoring via the WeChat Mini Program “Xunkang Assessment System” 3 times daily for 14 days. The compliance rate was calculated as the percentage of completed questionnaires out of the total required over 2 weeks. Multivariate ordinal logistic regression was used to explore the factors associated with the compliance rate.

**Results:**

The median compliance rate was 75% (IQR 35.7%-90.4%). Compliance did not differ significantly across mood states (*P*=.15). In multivariable models, higher Bech-Rafaelsen Mania Scale scores and lower Functioning Assessment Short Test scores were independently associated with better compliance (Bech-Rafaelsen Mania Scale: B=0.11; *P*=.03 and Functioning Assessment Short Test: B=−0.06; *P*=.01).

**Conclusions:**

Two-week EMA monitoring via the WeChat Mini Program is feasible among Chinese individuals with bipolar disorder across mood states. Manic symptom severity and functional impairment were associated with EMA adherence and should be considered in study design and interpretation.

## Introduction

Bipolar disorder manifests as depressive, manic, or hypomanic mood swings, often accompanied by intraday fluctuations [1]. The unpredictable nature of bipolar episodes contributes to recurrent and chronic illness, impairing emotional regulation, cognitive functioning, and social role performance, while placing a significant burden on public health systems. According to the Global Burden of Disease 2019 study, the global disability-adjusted life years attributable to bipolar disorder increased by approximately 54% between 1990 and 2017—rising from 6.02 million to 9.29 million [2]. Moreover, these mood fluctuations present considerable challenges for early detection and timely intervention [3].

Traditional face-to-face assessments, conducted infrequently and reliant on patient recall, struggle to capture real-time symptom changes [4,5]. The ecological momentary assessment (EMA) [6] can address these limitations by collecting real-time data in naturalistic settings [7]. EMA has been used to characterize symptoms, identify relapse indicators and track their progression, and monitor treatment effects [8,9]. Furthermore, a recent meta-analysis of 14 randomized controlled trials (n=1776) reported that EMA-based interventions significantly reduced bipolar relapse and improved medication adherence [10]. Earlier EMA studies often relied on paper-and-pencil methods or study-specific devices, which increased participant burden [7]. With the widespread adoption of smartphones, participants can integrate assessment into daily routines, facilitating the collection of individual-level phenotypes and reducing burden compared with paper methods. Smartphones also enable automatic logging of response times, improving data reliability and precision [11].

However, the main challenge in using EMA is patient compliance [9,12]. Studies have shown that response rates to paper-and-pencil EMA surveys in psychosis, depression, and healthy participants ranged from 66% to 93% [13]. Two smartphone-based EMA studies in patients with bipolar disorder reported compliance rates of 80.4% in 21 days among adolescents and young adults aged 14 to 21 years [14] and 70% in 14 days among adults aged 18 to 65 years [15]. In contrast, few EMA studies have been conducted in China. One study conducted in Hong Kong found that only 70.8% of patients with bipolar disorder completed one-third of entries in 6 consecutive days [16], suggesting potentially lower compliance among Chinese participants than in Western contexts.

Cultural and systemic factors may further affect EMA engagement in China. Cultural emphasis on saving face and pronounced stigma surrounding mental illness can lead to concealment of mood fluctuations and delays in seeking care [17]. Limited recognition of treatment needs may also reduce motivation for sustained self-monitoring [18]. In addition, the expansion of digital mental health services has lagged behind that of many Western countries, with per-capita investment below the global average. This service gap may limit the adoption of digital health tools [19]. Digital EMA tools may overcome accessibility hurdles by enabling *mobile-as-a-service* through ecological momentary sampling, instantly scaling assessment nodes for the Chinese population [20].

However, few studies have examined EMA use in Chinese patients with bipolar disorder, and compliance rates and their correlates remain unclear. A previous study indicates that early adherence is a key precursor to long-term adherence [21]. Therefore, before deploying large-scale digital monitoring and intervention programs, it is important to evaluate EMA compliance and its predictors in this population. Previous research suggests that compliance rates may be affected by the form of study design, the demographic characteristics of samples, the participant population, and clinical features [6,14,22]. For example, greater current mood elevation symptom severity and a history of suicide attempts predicted considerably worse adherence in young individuals with bipolar disorder [14]. On the basis of these findings, it was hypothesized that greater symptom severity and lower functional status would be associated with lower EMA adherence. This study addressed two questions: (1) what is the level of EMA compliance among Chinese patients with bipolar disorder and (2) how is compliance associated with demographic and clinical characteristics, including symptom severity and functional status?

## Methods

### Participants

Participants were recruited from the outpatient clinic of Tianjin Anding Hospital from January 2023 to April 2024. The inclusion criteria were as follows: (1) age 18 years or older; (2) proficiency in using a smartphone; and (3) a diagnosis of bipolar I or bipolar II disorder according to the Diagnostic and Statistical Manual of Mental Disorders, Fourth Edition (DSM-IV) criteria. Participants with visual, hearing, intellectual, or physical disabilities; schizophrenia spectrum or other psychotic disorders; mixed episodes of bipolar disorder; substance use disorders; severe physical illnesses; and who were pregnant were excluded.

Participants were interviewed by trained psychiatrists using the Structured Clinical Interview for DSM-IV Disorders, Research Version (SCID-IV-RV) [23]. Eight psychiatrists with more than 2 years of clinical psychiatric experience underwent a rigorous 10-day Structured Clinical Interview for DSM-IV Disorders (SCID) training program and successfully passed the examination to ensure their proficiency with the diagnostic criteria. Standardized quantitative record forms and audio recordings were used to document the diagnostic information of each participant in detail. For each patient, 2 psychiatrists independently conducted diagnostic evaluations; if there were differences in the initial diagnoses, a senior supervisor conducted a joint discussion to reach a final diagnosis. The information about demographic and clinical characteristics was also collected. Meanwhile, the severity of symptoms and functional impairment were assessed using the 17-item Hamilton Depression Rating Scale (HAM-D17) [24], the Bech-Rafaelsen Mania Scale (BRMS) [25], and the Functioning Assessment Short Test Scale (FAST) [26].

After the baseline assessment, participants were divided into three groups based on the HAM-D17 and BRMS scores: (1) depressive episode group (total HAM-D17 score³17 points and total BRMS score£5 points), (2) current hypomanic or manic episode group (total BRMS score>5 points and total HAM-D17 score<17 points), and (3) euthymic state group (total HAM-D17 score<17 points and total BRMS score≤5 points). All participants were invited to complete a 2-week self-monitoring period.

### EMA Procedure

**Figure 1:**
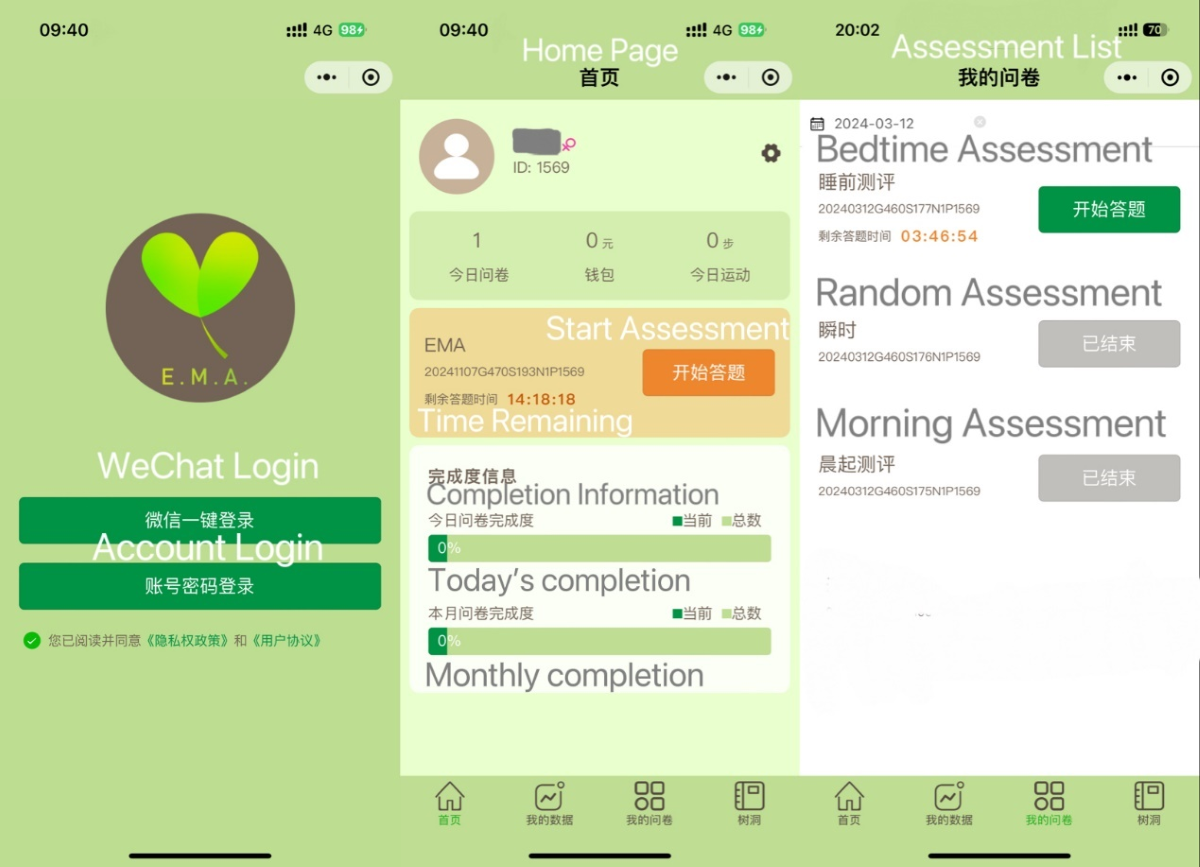
Screenshot of the interface of the Xunkang Assessment System.

All participants who completed the baseline survey were invited to register and conduct daily active self-monitoring via the WeChat Mini Program “Xunkang Assessment System” (Guangzhou Kangda Technology Co, Ltd), as shown in Figure 1. The WeChat Mini Program was designed to simplify responses through one-click access, a minimal interface (3 sliders and 1 submission button per page), and features to accommodate varying levels of digital literacy. The response data were encrypted and uploaded to the cloud in real time to ensure data security (for details regarding the user privacy policy, please refer to Multimedia Appendix 1). Typically, participants were prompted to complete questionnaires 3 times per day, including 2 scheduled questionnaires and a random questionnaire. The morning questionnaire was sent at 9 AM, the evening questionnaire at 8 PM, and the random questionnaire was dispatched at any time between 9 AM and 9 PM. Two participants required timing adjustments due to personal schedules: the evening questionnaire was shifted to 9 PM for 1 male participant (morning and random questionnaires unchanged), and the morning questionnaire was shifted to 08:30 AM for 1 female participant (random and evening questionnaires unchanged). For all participants, each questionnaire had a 90-minute response window. Participants who did not complete the questionnaire in time received a reminder every 30 minutes after the initial notification.

Literature indicates that monetary incentives are regarded as an “external reinforcement” mechanism in EMA research, aimed at enhancing participant completion rates. Systematic reviews and meta-analyses have demonstrated that EMA studies incorporating monetary incentives frequently report higher overall compliance rates [27]. However, multiple large-scale reviews have found no significant differences in the efficacy of monetary compensation for improving participant compliance across various emotional disorders, such as depression and anxiety [28]. This suggests that monetary incentives alone exert relatively balanced “enhancement” effects across different emotional states.

### Instruments

#### Demographic and Clinical Characteristics

Baseline information was collected, including gender, age, living area, years of education, employment status, illness duration, marital status, family history of mental illness, general physical condition, health beliefs regarding medication, stigma related to mental illness, suicidal ideation, level of self-insight, and alcohol and tobacco use.

#### Diagnosis of Psychiatric Disorders

The SCID-IV-RV [23] is a semistructured interview for the assessment and diagnosis of psychiatric disorders. It was designed to assist clinicians and researchers in systematically assessing psychiatric disorders according to DSM-IV criteria. The SCID-IV-RV consists of multiple modules, each corresponding to a specific category of mental disorder. The diagnoses applicable to SCID-IV-RV include (1) mood disorders, (2) psychotic disorders, (3) substance use disorders, (4) anxiety disorders, (5) somatoform disorders, (6) eating disorders, and (7) adjustment disorders.

#### Severity of Depression

HAM-D17 [24] was used to assess the severity of a participant’s depression over the past week. The scale includes 17 items that cover a variety of aspects, such as mood, somatic symptoms, cognitive symptoms, and daily activities. Each item was scored according to the severity of the symptoms, with scores ranging from 0 to 4 or 0 to 2. Higher total scores indicate more severe depressive symptoms, with conventional severity thresholds of mild (8-17), moderate (18-24), and severe (≥25). The Chinese version of the HAM-D17 has shown strong interrater reliability (κ=0.92) and acceptable internal consistency (Cronbach α= 0.714), with good structural validity [29].

#### Severity of Manic Symptoms

The BRMS [25] was used to assess the severity of manic symptoms in participants over the past week. The BRMS consists of 11 items covering elevated mood, speech activity, decreased sleep, difficulty concentrating, and impulsive behavior. Each item in the BRMS uses a 5-level scoring scale of 0 to 4. Each item has a standard scoring rule, and the total score is the primary outcome measure used for assessment. A total score of 0 to 5 indicates no significant manic symptoms, 6 to 10 indicates the presence of manic symptoms, and a score of 22 or more indicates that the patient has severe manic symptoms. The Chinese version of the BRMS has demonstrated excellent interrater reliability, with an intraclass correlation coefficient ranging from 0.97 to 0.99, and good construct validity, with a correlation coefficient of 0.92 when compared with the Global Assessment Scale [30].

#### Function Assessment

The FAST [26] was administered to assess the functioning of participants during the last 2 weeks. The FAST consists of 24 items divided into 6 functional domains: autonomy, occupational functioning, cognitive functioning, financial problems, interpersonal relationships, and leisure activities. Each item was scored according to the severity of functional impairment on a scale of 0 to 3, with a total score ranging from 0 to 72. Scores of ≤11 indicate no functional impairment, 12 to 20 indicate mild functional impairment, 21 to 40 indicate moderate functional impairment, and >40 indicate severe functional impairment. A higher total score indicates more severe functional impairment. The Chinese version of the FAST has demonstrated high internal consistency (Cronbach α=0.89 at baseline and 0.88 at week 1) and strong concurrent validity, as evidenced by significant correlations with the Global Assessment of Functioning scores and other established scales [31].

#### EMA Questionnaires

The daily self-assessment questionnaire included 3 self-assessments per day, including morning assessment, random assessment, and bedtime assessment. In the morning self-assessment, participants were required to report on several sleep metrics from the previous night, including sleep duration, time in bed, and subjective evaluation of sleep quality. Random self-assessment involved monitoring the participant’s current mood, energy level, cognitive functioning, and activity level. For the bedtime self-assessment, the participants were asked to report their midday nap that day, tobacco or alcohol use, any life event that affects participant’s emotions, and overall activity level throughout the day. For items in the daily self-assessment questionnaire, refer to Multimedia Appendix 2.

This study did not conduct a systematic statistical analysis of the collected EMA data because its primary objective was to predict adherence based on participants’ clinical and demographic characteristics before the EMA project’s commencement. The acceptability assessment in this study referenced findings from our prior in-depth interviews with participants, using a semistructured interview guide and conducting content analysis of the themes [32]. The data gathered through EMA served as core research material accumulated for subsequent studies exploring dynamic affective patterns and relapse prediction in patients with bipolar disorder.

### Statistical Analysis

The differences in demographic and clinical parameters between groups were analyzed. Chi-square tests were used for categorical variables, and the Kruskal-Wallis *H* tests were used for continuous variables. The compliance rate was defined as the number of completed monitoring sessions divided by the total number of scheduled monitoring sessions over the 2-week period. Full adherence was defined as the completion of a total of 42 self-monitoring sessions (3 times per day), conducted by each participant over a period of 2 weeks [14]. To examine the trajectory of EMA adherence over time, a linear mixed-effects model was fitted to daily adherence rates. On the basis of prior research and the minimum data requirements for EMA analysis, participants were categorized into 3 groups according to their compliance rates: poor compliance (0%≤X<30%), insufficient compliance (30%≤X≤80%), and adequate compliance (80%<X≤100%). These thresholds were adopted from previous studies [14,16,33,34] to ensure consistency and interpretability [35]. Fisher exact test was used to compare the differences in adherence rates between different mood state groups. To investigate the demographic and clinical factors associated with the compliance rate, univariate ordered logistic regression analysis was conducted. In total, 11 candidate factors (5 demographic variables and 6 clinical parameters) were evaluated in the univariable screen. Variables achieving *P*<.20 in univariate ordered logistic screening (5 clinical predictors) were entered into the final multivariate model [36]. The proportional odds assumption was upheld (score test *χ*^2^_5_=8.8; *P*=.12), justifying the use of ordinal logistic regression (PLUM, SPSS software; version 26.0; IBM Corp). All statistical analyses were performed in SPSS software.

This observational study was conducted in accordance with the STROBE (Strengthening the Reporting of Observational Studies in Epidemiology) guidelines.

### Ethical Considerations

This study adhered to the fundamental principles of the Declaration of Helsinki and was reviewed and approved by the Ethics Committee of Tianjin Anding Hospital (2022-56). Informed consent was obtained from all participants before their involvement in this study. All research data presented were also deidentified. In this study, all participants who completed the baseline assessment were informed that they would receive 100 RMB (US $14.13) upon completion of the 2-week dynamic active self-monitoring period.

## Results

A total of 100 participants diagnosed with bipolar disorder were recruited for this study, including 17 (17%) patients with a current manic or hypomanic episode, 54 (54%) with a euthymic state, and 29 (29%) with a depressive episode. The demographic characteristics and between-group comparisons are presented in Table 1. During the 14-day dynamic monitoring period, no patients withdrew from the study.

**Figure 2:**
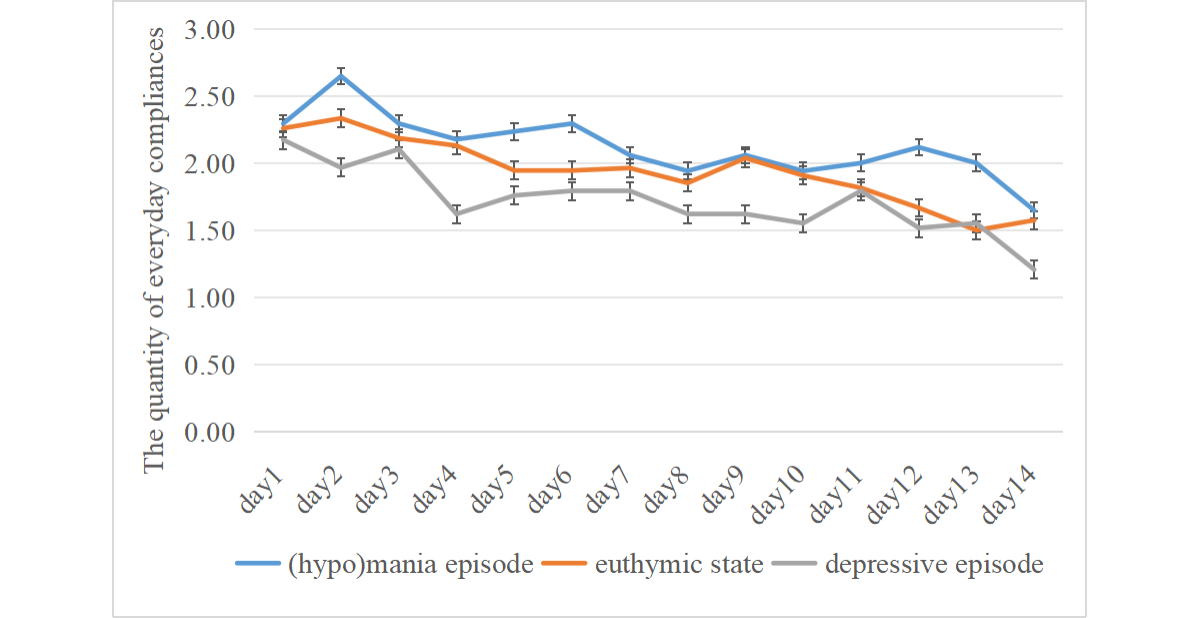
The quantity of everyday compliances in different mood state groups.

The median compliance rate for all samples was 75% (IQR 35.7%-90.4%). The compliance rate was 69.1% (IQR 45.1%-94.0%) for the hypomanic or manic episode group, 66.7% (IQR 34.5%-82.1%) for the depressive episode group, and 78.6% (IQR 33.3%-91.1%) for the euthymic group. There was no statistically significant difference among the compliance rates of the 3 groups (*P*=.15). This nonsignificant finding reflects insufficient evidence to reject the null hypothesis of equal compliance, rather than confirming that no true difference exists. The Fisher exact test results are shown in Table 2. Although the hypomanic or manic episode group showed a numerically higher adherence rate, this difference did not reach statistical significance and therefore warrants cautious interpretation. During the 2-week period, the hypomanic or manic group completed more morning (10 times) and evening assessments (10 times) than random prompts (9 times), whereas the depressive episode group (9 times at each time point) and euthymic group (8 times at each time point) completed equal numbers across all 3 time points. Daily completion trajectories over 2 weeks are shown in Figure 2, indicating declines over time in all groups. Mixed-effects analysis revealed a significant group×time interaction (*P*<.001). Specifically, the hypomanic or manic episode group declined most rapidly (slope −0.03, 95% CI −0.07 to −0.03), followed by the euthymic group (slope −0.02) and the depressive episode group (slope −0.02; Table 3).

Table 4 presents the results of the univariate analysis and the demographic and clinical characteristics corresponding to different compliance categories. In the multivariate model, illness duration (years), medication adherence, total HAM-D17 score, total BRMS score, and total FAST score were included. The final model showed low-to-moderate explanatory power (Nagelkerke *R*²=0.16; Akaike information criterion=208.4), implying that baseline demographics and clinical variables alone provided some insight into 14-day EMA compliance but were not highly predictive. Each 1-point increase in BRMS total score was associated with a 12% increase in the odds of higher adherence (odds ratio [OR] 1.12, 95% CI 1.01-1.24), whereas each 1-point increase in FAST score was associated with a 6% decrease in odds (OR 0.94, 95% CI 0.90-0.99). These results indicate that BRMS and FAST provide modest but meaningful predictive information for early identification of nonadherent individuals. Full results are presented in Table 5.

**Table 1:** Sociodemographic and clinical characteristics of different groups (N=100).

Variables			Hypomanic or manic episode (n=17)		Euthymic state (n=54)		Depressive episode (n=29)		H/chi-square (*df*)		*P* value	
Female, n (%)			10 (58.8)		32 (59.3)		21 (72.4)		1.6 (2)		.46	
Age (y), median (IQR)			25.0 (19.5-32.5)		27.0 (22.0-37.0)		23.0 (20.5-25.5)		5.26 (2)		.07	
**Marital status, n (%)**									2.9 (2)			.23
	Married	5 (29.4)		21 (38.9)		6 (20.7)						
	Unmarried	12 (70.6)		33 (61.1)		23 (79.3)						
**Education (y), n (%)**									0.9 (2)			.64
	<9	5 (29.4)		12 (22.2)		9 (31)						
	>9	12 (70.6)		42 (77.8)		20 (69)						
**Employment, n (%)**									3.4 (2)			.18
	Full-time job	7 (41.2)		28 (51.9)		9 (31)						
	Non–full-time job	10 (58.8)		26 (48.1)		20 (69)						
Illness duration (year), median (IQR)			4.0 (0.8-9.5)		3.0 (1.0-6.3)		4.0 (2.0-6.0)		0.41 (2)		.81	
Medication adherence, median (IQR)			2.0 (1.0-3.0)		2.0 (1.0-3.0)		2.0 (0-3.0)		0.51 (2)		.78	
**Suicidal ideation, n (%)**									0.8 (2)			.66
	Yes	9 (52.9)		22 (40.7)		12 (41.4)						
	No	8 (47.1)		32 (59.3)		17 (58.6)						
FAST^a^ score, median (IQR)			22.0 (10.5-29.0)		11.5 (6.8-16.3)		20.0 (14.0-29.0)		24.55 (2)		*<.001* ^b^	
BRMS^c^ score, median (IQR)			8.0 (6.0-13.0)		1.0 (0.0-2.0)		1.0 (0.0-2.5)		43.78 (2)		*<.001*	
HAM-D17^d^ score, median (IQR)			7.0 (3.5-14.5)		8.5 (3.0-13.0)		19.0 (18.0-23.0)		61.49 (2)		*<.001*	
Compliance rates (%), median (IQR)			69.1 (45.1-94.0)		78.6 (33.3-91.1)		66.7 (34.5-82.1)		2.16 (2)		.34	

^a^FAST: Functioning Assessment Short Test Scale.

^b^Italicization indicates significant *P* values.

^c^BRMS: Bech-Rafaelsen Mania Scale.

^d^HAM-D17: 17-item Hamilton Depression Rating Scale.

**Table 2:** Compliance rates in different mood state groups (N=100).

Variables	Poor compliance (n=19), n (%)	Insufficient compliance (n=39), n (%)	Adequate compliance (n=42), n (%)	*P* value
Hypomanic or manic episode	1 (5.9)	8 (47.1)	8 (47.1)	.15
Euthymic state	12 (22.2)	16 (29.6)	26 (48.1)	—^a^
Depressive episode	6 (20.7)	15 (51.7)	8 (27.6)	—

^a^Not available.

**Table 3:** Daily change in ecological momentary assessment adherence across 3 groups (N=42)^a^.

Variables	Intercept a (95% CI)	Daily slope b (95% CI)	*t* (*df*)	*P* value
Hypomanic or manic episode	2.24 (2.12 to 2.35)	−0.03 (−0.07 to −0.03)	227.74 (36)	<.001
Euthymic state	2.31 (2.20 to 2.42)	−0.02 (−0.02 to −0.016)	131.28 (36)	<.001
Depressive episode	2.24 (2.12 to 2.35)	−0.02 (−0.02 to −0.012)	—^b^	—

^a^Mixed-effects model (depressive episode group as reference) with fixed effects group, day (centered), and group×day interaction; random effects include participant-specific random intercepts and random slopes for day (variance‑components). Random-effect covariance is unstructured for the intercept-slope pair; residuals use a diagonal matrix.

^b^Not available.

**Table 4:** Univariate ordered logistic regression analysis (N=100).

Variables^a^			Poor compliance (n=19)		Insufficient compliance (n=39)		Adequate compliance (n=42)		B		*P* value		OR^b^ (95% CI)
**Sex** **, n (%)**													
	Male	9 (47.4)		14 (35.9)		14 (33.3)		−0.36		.35		0.70 (0.33-1.49)	
	Female	10 (52.6)		25 (64.1)		28 (66.7)		—^c^		—		—	
Age (y), median (IQR)			25.0 (21.0-34.0)		24.0 (21.0-34.0)		25.0 (21.8-35.3)		−0.01		.68		0.99 (0.96-1.03)
Illness duration (years), median (IQR)			4.0 (2.0-5.0)		3.0 (1.0-6.0)		4.0 (1.8-7.5)		0.06		*.19* ^*d*^		1.06 (0.97-1.16)
**Marital status, n (%)**													
	Unmarried	10 (52.6)		29 (74.4)		29 (69.0)		0.340		.40		1.41 (0.63-3.13)	
	Married	9 (47.4)		10 (25.6)		13 (31.0)		—		—		—	
**Employment status, n (%)**													
	Non–full-time job	11 (57.9)		24 (61.5)		21 (50.0)		−0.32		.40		0.73 (0.35-1.53)	
	Full-time job	8 (42.1)		15 (38.5)		21 (50.0)		—		—		—	
**Education (y), n (%)**													
	≤9	6 (31.6)		8 (20.5)		12 (28.6)		0.05		.91		1.05 (0.45-2.46)	
	>9	13 (68.4)		31 (79.5)		30 (71.4)		—		—		—	
**Suicidal ideation, n (%)**													
	No	13 (68.4)		22 (56.4)		22 (52.4)		−0.40		.29		0.67 (0.32-1.41)	
	Yes	6 (31.6)		17 (43.6)		20 (47.6)		—		—		—	
Medication adherence^e^, median (IQR)			1.0 (0-2.0)		2.0 (1.0-3.0)		2.0 (1.0-3.0)		0.28		*.05*		1.32 (0.99-1.74)
HAM-D17^f^ score, median (IQR)			13.0 (6.0-18.0)		15.0 (5.0-18.0)		10.5 (4.0-16.0)		−0.04		*.16*		0.97 (0.92-1.01)
FAST^g^ score, median (IQR)			22.0 (15.0-23.0)		14.0 (8.0-23.0)		12.5 (7.0-17.5)		−0.04		*.02*		0.96 (0.92-0.99)
BRMS^h^ score, median (IQR)			0 (0-2.0)		2.0 (0-5.0)		2.0 (0.8-5.0)		0.06		*.21*		1.06 (0.97-1.16)

^a^The collinearity diagnosis of the independent variables showed that there was no collinearity. The reference values for the categorical independent variables were female, unmarried, non–full-time job, ≤9 years of education, and no suicidal ideation.

^b^OR: odds ratio.

^c^Not available.

^d^Italicization indicates significant *P* values.

^e^The medication adherence of patients is assessed using 4 questions, each with answers scored as “Yes=0 point” and “No=1 points.” According to the scoring criteria of the Morisky Green Levine Medication Adherence Scale [37], higher scores indicate poorer adherence. A score of 0 denotes poor adherence, 1 to 2 denotes moderate adherence, and 3 to 4 denotes good adherence.

^f^HAM-D17: 17-item Hamilton Depression Rating Scale.

^g^FAST: Functioning Assessment Short Test Scale.

^h^BRMS: Bech-Rafaelsen Mania Scale.

**Table 5:** Multivariate ordered logistic regression model.

Variables	B (SE)	*P* value	OR^a^ (95% CI)
BRMS^b^ score	0.11 (0.05)	.03	1.12 (1.01-1.24)
HAM-D17^c^ score	0.25 (0.03)	.44	1.03 (0.96-1.09)
FAST^d^ score	−0.06 (0.02)	.01	0.94 (0.90-0.99)
Illness duration (y)	0.05 (0.05)	.29	1.05 (0.96-1.16)
Medication adherence	0.29 (0.15)	.05	1.33 (1.00-1.78)

^a^OR: odds ratio.

^b^BRMS: Bech-Rafaelsen Mania Scale.

^c^HAM-D17: 17-item Hamilton Depression Rating Scale.

^d^FAST: Functioning Assessment Short Test Scale.

## Discussion

### Principal Findings

To the best of our knowledge, this is the first study to assess EMA compliance and its correlates in Chinese patients with bipolar disorder. More than half of the participants achieved a 2-week response rate exceeding 75%. At the current sample size, there was no statistically significant difference in 14-day EMA adherence across the 3 mood states. A decline in adherence was observed across all 3 mood state groups over the 2-week period. We used mixed-effects models to investigate the dynamic changes in EMA adherence over time and differences across clinical status groups. With the depressive episode group as the reference, the results revealed a significant interaction effect between group and time (*P*<.001), indicating statistically significant differences in the temporal decline of EMA adherence among the 3 clinical status groups. The hypomanic or manic episode group exhibited the steepest decline in adherence, with a slope of −0.03 (95% CI −0.07 to −0.03). This indicates that for every 10 days elapsed, the average adherence rate in this group is projected to decrease by approximately 0.3 units. Mild manic episodes represent a significant risk factor for accelerated decline in EMA adherence. This suggests that patients at this disease stage may require more proactive, individualized intervention strategies, such as simplified assessment processes, additional reminders, or motivational interviewing, to sustain engagement.

The overall compliance rate of 75% in this study is consistent with the 58% to 91.6% range reported in meta-analyses of EMA studies in bipolar disorder [38]. It was slightly lower than the adherence observed in adolescents and young adults with bipolar disorder (80.4%) [14] and comparable to the adherence reported in substance use populations using smartphone-based EMA (70.8%) [39]. In addition to differences in the samples, the methodology might also account for the compliance differences [40]. Previous studies have demonstrated that extending the response window can effectively improve EMA adherence rates [28]. The study used a 90-minute response window, compared with 120 minutes in the young-person cohort, which may have reduced participants’ opportunities to respond [14]. In addition, because the platform was a WeChat Mini Program, participants could mute or disable prompts, which may have lowered completion rates [34].

Among patients with bipolar disorder, hypomanic or manic symptom severity predicted EMA compliance; each unit increase in the BRMS score corresponded to higher self-monitoring adherence. Although statistically significant, this association exhibited a small effect size and has limited clinical significance when considered alone. For clarification, in univariate analysis, BRMS was treated as a categorical variable, whereas in multivariate analysis, BRMS was treated as a continuous variable, which exhibited higher sensitivity for association detection. Moreover, in univariate analysis, BRMS was not significantly associated with EMA adherence (*P*=.21). However, in the multivariate ordered logistic regression model, BRMS became significant (*P*=.03; OR 1.12). This shift revealed the presence of a suppression effect, indicating that the “masking” effect of FAST on the relationship between BRMS and adherence became apparent after model control [41]. Despite similar overall participation, manic symptoms drove self-monitoring: elevated mood and heightened motivation led patients in manic states to engage more frequently [1]. It should be noted that our findings primarily apply to outpatient individuals with preserved insight and digital capabilities. People with hypomania often maintain good social functioning and high self-motivation in daily life [23], providing objective conditions for sustained EMA use. The presence of individuals experiencing hypomania within the hypomanic or manic group may have enhanced overall adherence within this cohort. Additionally, the presence of intense energy and vitality associated with manic symptoms facilitates greater activity and participation in self-monitoring tasks. While there is no direct literature indicating that patients with bipolar disorder exhibiting manic symptoms have better compliance with EMA tasks, the core clinical features of mania, such as increased activities in daily life, suggest that these patients may approach daily tasks with higher levels of engagement and responsiveness [42]. However, impulsivity and novelty seeking during manic episodes may initially enhance adherence but can lead to a rapid decline due to subsequent distractibility [43]. One possible reason for this study’s outcome may be that the failure to differentiate between mild and severe manic symptoms obscured the internal negative effects, leaving only the positive contribution of mild mania.

This study found no significant association between depression severity (HAM-D17 scores) and EMA compliance rates in patients with bipolar disorder. While this result differed from the hypothesis, it is not an isolated finding and can be reasonably explained through multidimensional analysis. A meta-analysis demonstrated no significant difference in EMA adherence between individuals with depressive disorders and healthy participants [33]. A study examining depression, anxiety, and suicidal ideation further confirmed that baseline depression severity and symptom fluctuations during follow-up did not affect EMA completion rates [44]. Although 1 study noted a slight negative impact of depressive history on compliance, no differences were observed in compliance between patients currently experiencing depression and those in remission [40]. These findings suggest that depression-related symptoms may not be direct predictors of EMA adherence.

Functional impairment was inversely associated with adherence. Higher FAST scores predicted lower odds of better adherence, consistent with a systematic review linking greater functional impairment to reduced retention and completion in digital monitoring [38]. Because higher FAST scores indicate more severe impairment in daily functioning, participants with greater functional deficits showed poorer compliance over the 2-week self-monitoring period. This may be attributed to the fact that, although depressive and manic symptoms may not directly determine EMA adherence, the overall functional impairments they cause may indirectly reduce patients’ adherence to self-monitoring [45,46]. Future studies could use FAST subscales or targeted neuropsychological tests to isolate the independent impact of distinct functional domains on adherence. A second possibility is that, even in remission, residual depressive and manic symptoms continue to impair specific functional domains in patients with bipolar disorder [47]. Compared to healthy individuals, individuals with bipolar disorder experience greater cognitive, emotional, and technical burdens during the EMA task, as reflected by lower completion rates and higher subjective discomfort [15,48]. The FAST scale primarily assesses participants’ long-term functional limitations in daily living, cognition, and social functioning, whereas EMA adherence reflects short-term, situational behavioral responses to mobile prompts at specific time points. These measures differ fundamentally in their dimensions and temporal scope; thus, FAST scores should not be directly interpreted as the sole cause of poor EMA adherence. Therefore, the association between functional impairment and EMA compliance should be regarded as correlational rather than causal. It is also noteworthy that after incorporating relevant covariates into the multilevel model, the negative effect of FAST scores on EMA completion rates remained significant after controlling for related factors. This indicates that the association should be considered and measured as an important factor.

### Limitations

The study sample primarily consisted of patients with bipolar disorder treated at Tianjin Anding Hospital in Tianjin, China. Although the sample originated from multiple distinct regions, the findings remain limited in their generalizability to other psychiatric subgroups and geographic areas. Unfortunately, due to sample size limitations and imbalanced distribution within variables (such as living area, medication information, and family history), this study focused on exploring core clinical and demographic predictors of EMA adherence based on prior literature, while avoiding overfitting in multilevel analyses. The sample size for the hypomanic or manic group in this study was only 17, resulting in insufficient statistical power to detect differences in compliance across mood states. The findings of this study should not be generalized to individuals with bipolar mixed episodes and patients with bipolar disorder who are not proficient in using smartphones. Additionally, the findings reflect only short-term adherence patterns; long-term adherence requires further validation through extended observation periods in subsequent studies. Moreover, although we have provided full disclosure, due to potential tracking mechanisms on the WeChat platform, some participants may harbor concerns about data leakage, potentially leading to mild underreporting of self-reported emotional or behavioral data. The final model showed weak explanatory power, possibly due to unmeasured factors and potential nonlinear associations between predictors and adherence. The findings of this study can be cautiously extrapolated to a subset of patients with bipolar disorder undergoing EMA monitoring via WeChat Mini Programs. Additionally, to maintain consistency with previous research, this study used identical fixed adherence thresholds; this approach may introduce minor classification uncertainty for individuals at extremes of adherence. Furthermore, the 100 RMB incentive may have inflated adherence in some groups; its effect across mood states is unknown.

### Future Research Directions

This study, as a predictive investigation, examined the adherence characteristics of a subset of Chinese patients with bipolar disorder using WeChat Mini Programs for EMA monitoring. The generalizability of the findings is limited by single-center recruitment, a sample size, the cross-sectional study design, lack of consideration for seasonal effects, and population restrictions. While medication adherence was assessed, this study did not further examine the impact of specific drug classes or dosages on EMA adherence. The sample included a large number of drug types (≥10) and frequent polypharmacy. Due to sample size limitations, reliable multivariate modeling was challenging. Future studies should examine whether specific mood stabilizers or antipsychotics modulate EMA adherence in larger or more homogeneous cohorts. Additionally, future research could use stratified randomization of incentives within mood state strata to assess differential effects on adherence and should replicate findings across multicenter settings, regional populations, and individuals with diverse mental health conditions to enhance generalizability, including examining differences in EMA adherence between the bipolar subtypes and conducting regular clinical assessments to determine the validity of the collected EMA data.

### Conclusions

Two-week EMA monitoring via WeChat Mini Program is feasible among Chinese individuals with bipolar disorder across mood states. The observed decline in daily self-monitoring task completion over 2 weeks highlights the need for further investigation into the temporal dynamics of EMA compliance. Notably, greater manic symptom severity and greater functional impairment were associated with lower adherence, highlighting the importance of exploring diverse clinical features to better understand factors influencing EMA compliance.

## Appendix

Multimedia Appendix 1 Xunkang assessment system privacy policy and supporting statistical analysis.

Multimedia Appendix 2 Daily self-assessment questionnaire.

## Abbreviations

BRMS: Bech-Rafaelsen Mania Scale
EMA: ecological momentary assessment
FAST: Functioning Assessment Short Test
HAM-D17: 17-item Hamilton Depression Rating Scale
OR: odds ratio
SCID-IV-RV: Structured Clinical Interview for DSM-IV Disorders, research version
STROBE: Strengthening the Reporting of Observational Studies in Epidemiology
