# Supplementary material for: Demographic and Clinical Characteristics Influencing Ecological Momentary Assessment Compliance in Individuals With Bipolar Disorder: Observational Study
Source: JMIR Form Res. 2026 Jan 7;10:e74223. doi: 10.2196/74223 (PMC12824568; doi:10.2196/74223)
Supplement: Multimedia Appendix 1 [file formative_v10i1e74223_app1.docx]

- Xunkang Assessment System Privacy Policy

Ⅰ、 Introduction

1. This "Xunkang Assessment System Privacy Policy" (hereinafter referred to as "the Privacy Policy") applies to all products and services of Xunkang Assessment System, including iOS and Android applications and all terminal clients.

Important Notice: As Xunkang Assessment System offers a wide range of products and services, the privacy policy provided here applies to all offerings. For specific products/services, Xunkang Assessment System will establish separate privacy policies. You must fully read and agree to the entire content of each specific policy before using the product/service.

2.

Before using any products or services of Xunkang Assessment System, please carefully read and fully understand all the contents of this Privacy Policy. Once you use or continue to use the products/services of Xunkang Assessment System, you agree to our use and processing of your information in accordance with this Privacy Policy.

3.

We may update this Privacy Policy from time to time in accordance with laws, regulations, or business adjustments. When changes are made, we will notify you of the updated content through prominent prompts, push notifications, or messages after the version is updated.

4.

You acknowledge that we will only collect, use, process, and store your personal information under the revised Privacy Policy of Xunkang Assessment System after your confirmation and consent. You have the right to refuse to accept the revised Privacy Policy. Please note that refusing to accept it may result in your inability to fully utilize Xunkang Assessment System's services and features, or to achieve the intended service outcomes.

Ⅱ、 About Us

1. Xunkang Assessment System is provided with product operations and services by Guangzhou Kangda Technology Co., Ltd. (hereinafter referred to as "Kangda Technology").

3. The basic information of our main operating entity is as follows: Guangzhou Kangda Technology Co., Ltd., established on December 14,2021, with the registered address at Room G300,499, Yanling Road, Tianhe District, Guangzhou, and the usual office address at Room G300,499, Yanling Road, Tianhe District, Guangzhou.

Ⅲ、 explanation of nouns

Unless otherwise agreed, the terms used in this Privacy Policy are generally defined as follows:

1. Personal Information (as defined in GB/T 35273-2020 "Information Security Technology-Personal Information Security Specification"): refers to various electronically or otherwise recorded data that can identify a specific natural person individually or in combination with other information, or reflect the activities of a specific natural person. The personal information covered in this Privacy Policy includes: basic personal data (including name, date of birth, gender, address, phone number, email address); personal identification information (including ID card number); personal biometric information (including fingerprints, facial features); network identity identifiers (including system account, IP address, digital certificate); personal asset information (including bank account, password, transaction/consumption records, virtual currency, virtual transactions, redemption codes); contact list information; personal browsing history (including website visits, software usage records, active software lists); personal device information (including hardware serial number, model, MAC address, operating system type, software lists, unique device identifiers); and personal location information (including approximate geographical location, precise positioning data). The specific types of personal information we collect are as described below.

2. Personal Sensitive Information (as defined in GB/T35273-2020 "Information Security Technology-Personal Information Security Specification"): This refers to personal information that, if leaked, illegally disclosed, or misused, may endanger personal safety and property, or easily result in reputational damage, physical/mental health harm, or discriminatory treatment. The sensitive information covered in this Privacy Policy includes your personal financial information, identity details, biometric data, online identity identifiers, contact lists, precise location data, and delivery addresses. The specific types of sensitive information we collect are as described below.

3. Device: means a device that can be used to access our products and/or services, such as desktop computers, laptops, tablets, or smartphones.

4. Unique Device Identifier (UDI): (Exclusive ID or UUID) refers to a string of characters embedded by the device manufacturer into the device, which uniquely identifies the device (e.g., IMEI, Android ID, IDFA, OpenUDID, GUID, SIM card IMSI). UDI has multiple uses, including advertising when cookies are unavailable (e.g., in mobile applications).

5.IP address: Every device connected to the internet is assigned a unique number called an Internet Protocol (IP) address. These numbers are typically assigned based on geographical regions. IP addresses can be used to identify the location of a device when it connects to the internet.

6. SSL: SSL (Secure Socket Layer), also known as the Secure Socket Layer, is a security protocol built upon the TCP/IP communication protocol. It supports multiple network types and provides three core security services, all utilizing public and symmetric key technologies to ensure information confidentiality.

7. Cookies: Cookies are small files containing strings that are sent and stored on your computer, mobile device, or other device (typically encrypted) when you log in or use a website or other online content. Similar technologies include Web Beacons, proxies, and embedded scripts, which serve similar purposes.

8. Account: By registering and providing personal information, you gain better access to our services. When you visit Xunkang Assessment System System, the system uses this account information to verify your identity and prevent unauthorized access.

9. Anonymization: refers to the process of technical processing of personal information, so that the subject of personal information can not be identified or associated, and the processed information can not be restored.

10. De-identification: refers to the process of technical processing of personal information so that it cannot identify or associate the subject of personal information without additional information.

11. Server logs: Our servers typically automatically record web requests when you visit the site. These "server logs" usually include your network requests, IP addresses, browser type, browser language, request date and time, and one or more cookies that uniquely identify your browser.

IV. How We Collect and Use Your Personal Information

1. Xunkang Assessment System collects and uses your personal information in accordance with laws and regulations, adhering to the principles of legitimacy, legality, and necessity. This includes data you voluntarily provide during service usage or information generated through product/service requirements. Should we need to use your personal information for purposes not specified in this Privacy Policy, or to achieve specific objectives, we will promptly notify you through reasonable means and obtain your consent again before proceeding.

2. We collect and use two types of personal information about you:

Type 1: Essential Information for Core Business Functions: This refers to information required for the normal operation of our products/services. You must authorize us to collect this data. Failure to provide it will prevent you from using our products/services. Type 2: Information Required for Additional Business Features: This information is not essential for core business functions. You may choose whether to authorize its collection. Refusal to provide this information may result in the inability to implement additional features or achieve intended outcomes, but it will not affect your access to core business functions.

3. Please note: As our product and service offerings vary, the core business functions (including the types of personal information collected) may differ depending on the specific product/service. The actual features provided will be subject to the specific offerings. Additionally, you acknowledge and agree that we strive to continuously improve our products/services, including technical enhancements, which may involve introducing new business functions, collecting additional personal information, or modifying the purposes and methods of personal information usage. If any function or service requiring personal information collection is not explicitly covered in this Privacy Policy, we will provide additional information regarding the purpose, scope, and usage of such collection through policy updates, page prompts, pop-ups, or website announcements. You will have the option to choose your consent method, and any collection will only proceed after obtaining your explicit consent.

In this process, if you have any questions about the relevant matters, you can contact us through the methods provided in Article 11 of this Privacy Policy, and we will answer your questions as soon as possible.

4. We typically collect and use your personal information in the following scenarios:

4.1 Account Registration

(1) To register and log in to the Xunkang Assessment System account, you must complete the registration process as instructed. During this process, you will need to provide the following information: account nickname, mobile phone number, or email address (personal sensitive information used to verify your identity through verification codes). Only by providing accurate and truthful information can you successfully register for the Xunkang Assessment System account and access core product and/or service features. If you choose not to provide this essential information required for core product functionality, we may be unable to provide those core features.

(2) You should also understand that the matching results of your phone number or email address with the verification code are your sensitive personal information. We collect such information based on relevant legal and regulatory requirements. If you refuse to provide it, you may not be able to register an account or use the relevant product features. Please consider carefully before providing it.

4.2 Use Bluetooth devices and interactive mini-games at offline exhibitions

(1) When you use Bluetooth devices and enable Bluetooth, we may collect the current Bluetooth device connection status of your device, and provide Bluetooth headset volume adjustment, control video playback and pause, control video recording and pause, like video, monitor Bluetooth device connection status, and discover the functions of surrounding devices during face-to-face drinking activities. (1) When you use Bluetooth devices and enable Bluetooth, we may collect the current Bluetooth device connection status of your device, and provide Bluetooth headset volume adjustment, control video playback and pause, control video recording and pause, like video, monitor Bluetooth device connection status, and discover the functions of surrounding devices during face-to-face drinking activities.

Please understand that the connection status of a standalone Bluetooth device cannot identify an individual's identity. When such non-personal information is combined with other data to identify a specific individual, or when it is used in conjunction with personal information, it will be treated as personal information during the combined use period. Unless authorized by you or otherwise required by law, we will anonymize and de-identify this personal information.

(2) When you attend the BilibiliWorld offline event and turn on Bluetooth, we will provide exhibition tasks (such as exploring and capturing UP creators around you) and interactive games. To do this, we need to collect your Bluetooth connection status and scan the surrounding Bluetooth list.

Please understand that individual Bluetooth connection status and peripheral Bluetooth lists cannot identify specific individuals. When such non-personal information is combined with other data for identity verification, or integrated with personal information, it will be treated as personal information during the combined usage period. Unless authorized by you or otherwise required by law, we will anonymize and de-identify this personal information.

(3) If you do not wish to use the aforementioned features, you may enable or disable Bluetooth at any time through the relevant settings page on your device. Activating Bluetooth on your device will be deemed as granting us access to, retrieval of, collection of, and use of your personal information. However, when you disable Bluetooth, we will cease collecting such information and cannot provide the corresponding services. Unless you delete your personal information in accordance with legal requirements, your cancellation will not affect our processing and storage of your personal information based on prior authorization. Additionally, if you use the iOS device with iOS13.1 or later, you may also enable or disable this permission through the relevant settings page of our products/services. For the iOS version of the XunKang Evaluation APP, the Siri usage permission settings path is: "My Settings> Privacy Settings> Allow XunKang Evaluation to access Bluetooth". After updating the app version, we will not change your previously set permission status without your explicit consent.

4.3 Your personal information collected indirectly from third parties

(1) You acknowledge and understand that we cannot access or obtain your personal information from external third parties (including but not limited to affiliates of Xunkang Assessment System System) through any unauthorized means, such as malicious interference with their app data.

Xunkang may need to collect your personal information indirectly from third parties (e.g., through sharing) due to business development needs. When we directly or jointly provide products or services to you, we (or third parties) will clearly disclose the source, type, purpose, method, business functions, and authorized scope of the shared personal information before collection (if the usage method or scope exceeds your original authorization from the third party, we will seek your authorization again). For certain products or services provided by third-party business partners, we may indirectly collect partial information from some business partners or information related to you provided by other parties when using our products and/or services, to ensure necessary and reasonable business operations.

(2) Our professional security team will implement robust safeguards for personal information, including reporting sensitive data, encrypted storage, and access control. We will protect indirectly obtained personal information using no less stringent measures than those applied to our own users.

4.4 For other reasonable and necessary purposes

(1) As previously stated, if a feature or product/service requiring personal information collection is not specified in this Privacy Policy, or if our collection exceeds the stated purpose and directly or reasonably related scope, we will provide additional notice through updated Privacy Policy, page prompts, pop-ups, in-site messages, website announcements, or other accessible methods before collecting and using your personal information. We will offer you the option to consent through these methods and only collect and use your information after obtaining your explicit consent.

(2) You understand and agree that we may collect and use your personal information without your authorization and consent under the following circumstances:

a) Those related to national security and defense security;

b) those related to public safety, public health and major public interests;

c) directly related to criminal investigation, prosecution, trial and judgment execution;

d) For the purpose of safeguarding your or other individuals' life, property and other major legitimate rights and interests, but it is difficult to obtain your consent;

e) The information collected is either self-disclosed to the public or obtained from legally accessible sources (such as official news reports or government disclosures);

f) Necessary for the execution and performance of the relevant agreements or other written documents with you;

g) Necessary for maintaining the safe and stable operation of our products and/or services, such as the discovery and disposal of product and/or service failures;

h) Other circumstances stipulated by the requirements of competent authorities, laws and regulations, etc.

V. How We Share, Transfer, and Publicly Disclose Your Personal Information

1. Share

We attach great importance to the protection of your personal information. Your personal information is an important basis and component for us to provide you with products and/or services. We only collect and use your personal information for the purposes and scope described in this Privacy Policy or according to the requirements of laws and regulations, and strictly keep it confidential.

We do not normally share your personal information with any company, organization or individual, except as follows:

1.1 You have given your express authorization or consent in advance;

1.2 Sharing your personal information as required by applicable laws and regulations, legal procedures, government orders or judicial rulings;

1.3 To the extent permitted or required by law, it is necessary to provide your personal information to a third party to protect the interests, property or safety of Xunkang Assessment System System, its users or the public from damage;

2. Transfer

Unless you provide explicit consent, we will not transfer your personal information to any company, organization, or individual. In the event of a merger, acquisition, or bankruptcy liquidation involving personal information transfers, we will require the new entity holding your personal information to continue complying with this Privacy Policy. Should any changes occur to the collection or processing methods specified in this Privacy Policy, the company or organization will seek your authorization and consent again.

3. Public disclosure

3.1 Public disclosure refers to the act of releasing information to the public or unspecified groups. Except for necessary disclosures such as penalty announcements for non-compliant accounts, fraud cases, or the publication of winning/celebration lists with anonymized information, we will not publicly disclose your personal information. If there are reasonable grounds for disclosure, we will inform you of the purpose and type of information to be disclosed (including the specific content of sensitive personal information) before disclosure, and only proceed after obtaining your authorization and consent, unless otherwise stipulated by laws, regulations, or this policy.

3.2 For the personal information disclosed publicly by you, we will, as soon as possible and prudently, review the legitimacy, rationality and legality of the disclosure upon receiving the disclosure application, and take the strictest personal information security protection measures and means to protect the personal information when and after the disclosure.

4. Exceptions to sharing, transferring, or publicly disclosing your personal information

Please be advised that under the following circumstances, sharing, transferring or publicly disclosing your personal information does not require your prior authorization or consent in accordance with laws and regulations:

4.1

Those directly related to national security and national defense security;

4.2

Those directly related to public security, public health and major public interests;

4.3

Those directly related to criminal investigation, prosecution, trial and judgment execution; or those required by laws and regulations, administrative organs or competent authorities such as public security, procuratorate and court;

4.4

Where it is difficult to obtain your consent for the protection of your or other individuals' life, property and other major legitimate rights and interests;

4.54.5

Personal information refers to information that you disclose to the public or collect from legal public channels (such as legal news reports, government information disclosure, etc.);

4.6

As necessary to enter into and perform the relevant agreements or other written documents with you;

4.7

Other circumstances prescribed by laws and regulations.

VI. About Using Cookies and Similar Technologies

1. When using our products and/or services, we may collect certain personal information through cookies and similar technologies, including your browsing habits, browsing data, and login details. This information is collected to: enable seamless product/service usage, simplify repetitive steps (e.g., registration and login), provide usage history (e.g., video viewing history), deliver personalized services and content, protect your account security, and enhance our product and service quality.

2. If you refuse our use of cookies and similar technologies to collect and use your information, you can manage (partial or complete) refusal of cookies and/or similar technologies through your browser settings, provided your browser supports this feature. Alternatively, you may delete cookies and/or similar technologies stored on your computer, mobile device, or other devices, thereby preventing us from fully or partially tracking your personal information. For detailed instructions on changing browser settings, please refer to the relevant settings page of your browser.

You acknowledge and understand that certain products/services require cookies or similar technologies to function. If you disable or delete these technologies, you may not be able to use our products/services properly or enjoy optimal service experience. This may also affect your data protection and account security.

VII. Storage and Protection of Your Personal Information

1. Information storage

1.1 Storage Location: In accordance with applicable laws and regulations, we will only store your personal information within the territory of the People's Republic of China. Currently, we do not have any cross-border storage arrangements or overseas data transfer practices. If cross-border data transmission becomes necessary, we will provide you with a detailed notice specifying the purpose of data transfer, the receiving party, usage scope, content of data usage, security measures, and potential risks. We will obtain your explicit consent and ensure that the receiving party possesses adequate data protection capabilities to safeguard your personal information.

1.2 Retention Period: We retain your personal information for the shortest necessary period required for product and service purposes. For example, when you use our registration and membership features, we collect your phone number and retain it continuously during your usage to ensure proper functionality and account security. Additionally, we may retain your information for statutory periods as required by applicable laws (e.g., the E-Commerce Law stipulates that product/service and transaction information must be retained for at least three years from the transaction completion date).

1.3 After the above-mentioned storage period expires, we will delete or anonymize your personal information.

2. Information Protection

2.1 Your personal information security is of vital importance to us. We will strictly comply with relevant laws and regulations and take reasonable and feasible measures recognized by the industry to protect your personal information. We will prevent unauthorized access, disclosure, use, or modification of your information, and avoid damage or loss of your information.

2.2 We implement industry-standard security measures to protect your personal information, preventing unauthorized access, public disclosure, misuse, modification, damage, or loss. We take all reasonable and feasible steps to safeguard your personal data, including: 1) Using SSL encryption for data protection; 2) Establishing access control mechanisms to ensure only authorized personnel can access personal information; 3) Conducting regular security and privacy training sessions to enhance employees' awareness of the importance of personal information protection.

2.3 To prevent safety incidents, we have established a dedicated data security department and implemented robust early warning mechanisms and emergency response plans. In the event of a personal information security incident, we will promptly inform you in accordance with legal requirements: detailing the incident's basic circumstances and potential impacts, outlining the measures we have taken or will take, providing recommendations for risk mitigation, and explaining remedial actions available to you. We will immediately activate our emergency response plan to minimize losses. You will be notified of the incident through phone calls, push notifications, and other relevant channels.

VIII. Your Right to Manage Personal Information

We understand your concern about personal information and do our best to ensure your right to access, correct, delete, and withdraw authorization for your personal information, so that you have sufficient ability to protect your privacy and security. Your rights include:

1. You have the right to access your personal information

You may access the personal information you have provided to us in accordance with the instructions (or settings) provided by us for our products and services. This includes:

1.1 Account Information: You can log in to your personal center at any time through the product page to access your account information, including: avatar, nickname, UID, QR code business card, gender, birth date, personal signature, etc.

1.2 Usage information: You can check your usage information at any time on the relevant product page, including: dynamic content, submitted content, collection records, history records, order information, address information, billing records, etc.;

1.3 Other Information: If you encounter operational issues during your visit and need to obtain other personal information not covered herein, you may contact us through the methods specified in this Privacy Policy.

2. You have the right to correct or modify your personal information

If you find that the personal information you provide to us is incorrect, incomplete or updated, you may correct or modify your personal information in our products and/or services.

2.1 For some of your personal information, we provide operation settings on the product's relevant function pages. You can directly correct or modify it. For example, the correction path for "avatar/nickname/gender/birthdate/personal signature" in Xunkang Assessment System System's iOS client is: click Avatar-Edit Profile.

2.2 Regarding difficulties encountered while exercising the aforementioned rights, or other situations where we cannot currently provide online self-correction/modification services, you shall have the right to correct or modify erroneous or incomplete information after identity verification, provided that such corrections do not affect the objectivity and accuracy of the information. In specific circumstances, particularly when dealing with data errors, you may submit your correction/modification requests to us through published feedback mechanisms or error reporting channels, requesting us to rectify your data. Exceptions apply where otherwise stipulated by laws and regulations. However, for security and identity verification purposes, certain initial registration information submitted during registration may not be modified.

3. You have the right to delete your personal information

3.1 For certain personal information, you may also delete the data you provided through the functional pages of our related products and services. For example, in the Xunkang Assessment System iOS client, you can delete your "History" records via My-History-Edit. You may also independently delete videos, posts, images, and other content you have published. Once deleted, we will remove or anonymize such information unless otherwise required by law.

3.2 You may directly request us to delete your personal information when the following circumstances occur, except that the personal information has been anonymized or otherwise provided by laws and regulations:

(1) When we process personal information in violation of laws and regulations; (2) When we collect and use your personal information without your consent;

(3) When our processing of personal information violates the agreement with you;

(4) When you log out of the Xunkang Assessment System account; (5) When we terminate the service and operations.

4. You have the right to withdraw your authorization for personal information

As previously stated, certain features of our products and services require specific device permissions (including location, camera, microphone, calendar, etc.), as detailed in Article 4 of this Privacy Policy.

You may withdraw (or terminate) this permission at any time after granting it. When you update the App version, we will not change your previously set permission status without your explicit consent. For example, you can disable access to your phone's photo album through "Settings> Privacy> Photos" on your mobile device. You can also permanently revoke all authorization for us to continue collecting your personal information by signing out of your account. Please note that after withdrawing authorization, we will no longer provide the specific features and/or services associated with it. However, your decision to withdraw authorization will not affect any personal information processing previously conducted based on your prior authorization.

5. You have the right to cancel your account

You can cancel your account online, through customer service, or through other methods we have announced.

After account cancellation, you will no longer be able to log in or use our products/services with this account. All accrued but unused benefits and future entitlements generated during the account's use on Xunkang Assessment System and its affiliated services will be fully revoked. Content, information, data, and records under this account will be deleted or anonymized (unless otherwise required by laws or regulatory authorities, such as the Cybersecurity Law of the People's Republic of China, which mandates retaining your network operation logs for at least six months). Once an Xunkang Assessment System account is canceled, it cannot be restored.

If you still decide to cancel your Xunkang Assessment System account after careful consideration, you may contact us through the contact information provided to proceed with the cancellation.

We will respond to your request for account cancellation within 15 working days after verifying your identity. If you have any questions regarding the processing of your personal information, please contact us through the contact details provided in Section 11 "Contact Us" of this Privacy Policy.

IX. Our protection of minors

1. Minors shall obtain the consent of their guardians before using our products and/or services.

If you are a minor, you should read this Privacy Policy together with your guardian before using our products and/or services, and use our products and/or services and submit personal information with your guardian's explicit consent and guidance.

We protect the personal information of minors in accordance with the relevant laws and regulations of the state. We will only collect, use or disclose the personal information of minors when permitted by laws and regulations, with the explicit consent of the guardian or when necessary to protect your rights and interests.

2. If you are the guardian of a minor and have questions about their personal information, you may contact us through the contact details provided in Section 11 "Contact Us" of this Privacy Policy. Should we discover that we have collected personal information of minors without obtaining prior verifiable guardian consent, we will promptly delete the relevant data. X. We protect the personal information of deceased users.

1. Xunkang Assessment System will upgrade its deceased user data protection system in accordance with the Personal Information Protection Law starting November 1, 2021. After the death of a Xunkang Assessment System user (limited to natural persons), their close relatives may exercise rights to access, copy, correct, or delete the deceased user's personal information through the contact details provided in Section 11 "Contact Us" of this Privacy Policy for legitimate and proper interests. This applies unless the deceased user had made prior arrangements.

2. You acknowledge and confirm that, to fully protect the deceased user's personal information rights, the deceased user's close relatives applying for this right must submit the deceased user's identification documents, death certificate, the applicant's identification documents, and proof of kinship between the applicant and the deceased user, in accordance with Xunkang Assessment System's designated procedures or customer service instructions. Additionally, they must specify the type of right being exercised and the purpose of the application.

迅康测评隐私政策

Ⅰ、引言

1.本《迅康测评隐私政策》(以下称“本隐私政策”)适用于迅康测评的全部产品和服务，包括iOS端应用程序、安卓端应用程序等全部终端客户端。

特别提醒:由于迅康测评的产品和服务较多，为您提供的产品和服务内容也有所不同，本隐私政策为迅康测评统一适用的一般性隐私政策条款。针对迅康测评的某些特定产品/服务，迅康测评还将制定特定隐私政策，您应在充分阅读并同意特定隐私政策的全部内容后再使用该特定产品/服务。

2.

请您在使用迅康测评各项产品和/或服务前，仔细阅读并充分理解本隐私政策的全部内容。一旦您使用或继续使用迅康测评的产品/服务，即表示您同意我们按照本隐私政策使用和处理您的相关信息。

3.

我们可能会不时依据法律法规或业务调整对本隐私政策进行修订。当本隐私政策发生变更后，我们会在版本更新后通过在显著位置提示或推送通知、消息等形式向您展示变更后的内容。

4.

您需理解，只有在您确认并同意变更后的《迅康测评隐私政策》，我们才会依据变更后的隐私政策收集、使用、处理和存储您的个人信息;您有权拒绝同意变更后的隐私政策，但请您知悉，一旦您拒绝同意变更后的隐私政策，可能导致您不能或不能继续完整使用迅康测评的相关服务和功能，或者无法达到我们拟达到的服务效果。

Ⅱ、关于我们

1.迅康测评由广州康达科技技术有限公司(以下称“康达科技”)提供产品运营和服务。

3.我们的主要运营主体的基本信息如下:广州康达科技技术有限公司，成立于2021年12月14日，注册地址为广州市天河区燕岭路89号499室G300号，常用办公地址为广州市天河区燕岭路89号499室G300号。

Ⅲ、名词解释

除非另有约定，本隐私政策中使用到的名词，通常仅具有以下定义:

1.个人信息(出自于GB/T 35273-2020《信息安全技术个人信息安全规范》):是指以电子或者其他方式记录的能够单独或者与其他信息结合识别特定自然人身份或者反映特定自然人活动情况的各种信息。本隐私政策中涉及的个人信息包括自然人的基本资料(包括个人姓名、生日、性别、住址、个人电话号码、电子邮箱地址)、个人身份信息(包括身份证件号码)、个人生物识别信息(包括指纹、面部特征)、网络身份标识信息(包括系统账号、IP地址、个人数字证书等)、个人财产信息(包括银行账号、口令、交易和消费记录、虚拟货币、虚拟交易、兑换码等虚拟财产信息)、通讯录信息、个人上网记录(包括网站浏览记录、软件使用记录、使用中的软件列表)、个人常用设备信息(包括硬件序列号、硬件型号、设备MAC地址、操作系统类型、软件列表、唯一设备识别码)、个人位置信息(包括大概地理位置、精准定位信息)。我们实际具体收集的个人信息种类以下文描述为准。

2.个人敏感信息(出自于GB/T35273-2020《信息安全技术个人信息安全规范》):是指一旦泄露、非法提供或滥用可能危害人身和财产安全，极易导致个人名誉、身心健康受到损害或歧视性待遇等的个人信息。本隐私政策中涉及的个人敏感信息包括您的个人财产信息、个人身份信息、个人生物识别信息、网络身份标识信息、通讯录信息、精准定位信息、收货地址。我们实际具体收集的个人敏感信息种类以下文描述为准。

3.设备:是指可用于访问我们的产品和/或服务的装置，例如台式计算机、笔记本电脑、平板电脑或智能手机。

4.唯一设备识别码:(专属ID或UUID):是指由设备制造商编入到设备中的一串字符，可用于以独有方式标识相应设备(如 IMEI/android ID/IDFA/OpenUDID/GUID/SIM卡IMSI信息等)。唯一设备识别码有多种用途，其中可在不能使用Cookie (例如在移动应用程序中)时用以提供广告。

5.IP地址:每台上网的设备都会指定一个编号，称为互联网协议(IP)地址。这些编号通常都是根据地理区域指定的。IP地址通常可用于识别设备连接至互联网时所在的位置。

6. SSL:SSL(Secure Socket Layer)又称为安全套接层，是在传输通信协议(TCP/IP)上实现的一种安全协议。 SSL支持各种类型的网络，同时提供三种基本的安全服务，均通过使用公开密钥和对称密钥技术以达到信息保密的效果。

7.Cookie:Cookie是包含字符串的小文件，在您登入和使用网站或其他网络内容时发送、存放在您的计算机、移动设备或其他装置内(通常经过加密)。Cookie同类技术是可用于与Cookie类似用途的其他技术，例如:Web Beacon、Proxy、嵌入式脚本等。

8.账号:当您注册账号并向我们提供一些个人信息，您就可以更好的使用我们的服务。当您访问迅康测评时，系统会利用这些账号信息对您进行身份验证，以防止未经授权的人员访问您的账号。

9.匿名化:是指通过对个人信息的技术处理，使得个人信息主体无法被识别或者关联，且处理后的信息不能被复原的过程。

10.去标识化:是指通过对个人信息的技术处理，使其在不借助额外信息的情况下，无法识别或关联个人信息主体的过程。

11.服务器日志:通常情况下，我们的服务器会自动记录您在访问网站时所发出的网页请求。这些“服务器日志”通常包括您的网络请求、互联网协议地址、浏览器类型、浏览器语言、请求的日期和时间及可以唯一识别您的浏览器的一个或多个Cookie。

Ⅳ、我们如何收集和使用您的个人信息

1.迅康测评依据法律法规以及遵循正当、合法、必要的原则而收集和使用您在使用服务过程中主动提供或由于产品和/或服务需要而产生的个人信息。如果我们欲将您的个人信息用于本隐私政策未载明的其它用途，或基于特定目的将收集而来的信息用于其他目的，我们会及时以合理的方式向您告知，并在使用前再次征得您的同意。

2.我们收集和使用的您的个人信息类型包括两种:

第一种:我们产品与/或服务的核心业务功能所必需的信息:此类信息为产品与/或服务正常运行的必备信息，您须授权我们收集。如您拒绝提供，您将无法正常使用我们的产品与/或服务;第二种:附加业务功能可能需要收集的信息:此信息为非核心业务功能所需的信息，您可以选择是否授权我们收集。如您拒绝提供，将导致附加业务功能无法实现或无法达到我们拟达到的效果，但不影响您对核心业务功能的正常使用。

3.我们需要特别提醒的是:由于我们的产品和服务较多，为您提供的内容也不同，因此核心业务功能(包括其收集的您的个人信息类型)也会因产品/服务的内容不同而有所区别，具体以产品/服务实际提供为准。除此之外，您理解并同意，我们希望提供给您的产品和服务是完善的，所以我们会不断改进我们的产品和服务，包括技术，这意味着我们可能会经常推出新的业务功能，可能需要收集新的个人信息或变更个人信息使用目的或方式。如果某一需要收集您的个人信息的功能或产品/服务未能在本隐私政策中予以说明的，我们会通过更新本政策、页面提示、弹窗、网站公告等方式另行向您说明该信息收集的目的、内容、使用方式和范围，并为您提供自主选择同意的方式，且在征得您明示同意后收集。

在此过程中，如果您对相关事宜有任何疑惑的，可以通过本隐私政策第十一条提供的方式联系我们，我们会尽快为您作出解答。

4.通常情况下，我们会在以下场景中收集和使用您的个人信息:

4.1账号注册

(1)您注册并登录迅康测评账号时，需要按照我们的指引完成一系列注册程序，在此过程中，您需要向我们提供以下信息:账号昵称、手机号码或电子邮箱(个人敏感信息，用于接受验证码匹配个人身份)。您只有提供真实准确的上述信息，才能成功注册迅康测评账号并使用产品和/或服务的核心功能。如果您选择不提供上述为实现迅康测评核心产品功能的必备信息，或将导致我们无法为您提供该核心产品功能。

(2)同时，您需理解，手机号码或电子邮箱地址和验证码匹配结果属于您的个人敏感信息，我们收集该类信息是基于法律法规的相关要求，如您拒绝提供可能导致您无法注册账号并使用相关产品功能，请您谨慎考虑后再选择是否提供。

4.2使用蓝牙设备、线下展会互动小游戏

(1)当您使用蓝牙设备并开启蓝牙功能时，我们可能会搜集您的设备目前正在使用中的蓝牙设备连接状态，并提供蓝牙耳机音量调节、控制视频播放和暂停、控制视频录制和暂停、为视频点赞、监测蓝牙设备连接状态 面对面干杯活动发现周围设备功能。

请您理解，单独的蓝牙设备连接状态无法识别特定自然人的身份信息。如果我们将这类非个人信息与其他信息结合用于识别特定自然人身份，或者将其与个人信息结合使用，则在结合使用期间，这类非个人信息将被视为个人信息，除取得您授权或法律法规另有规定外，我们会将该类个人信息做匿名化、去标识化处理。

(2)当您参加BilibiliWorld线下展会并开启蓝牙功能时，我们会提供相应的展会任务(如探寻和捕获身边的UP主)以及互动游戏。为此，我们需要搜集您的蓝牙连接状态并扫描周围的蓝牙列表。

请您理解，单独的蓝牙连接状态、周围蓝牙列表无法识别特定自然人的身份信息。如果我们将这类非个人信息与其他信息结合用于识别特定自然人身份，或者将其与个人信息结合使用，则在结合使用期间，这类非个人信息将被视为个人信息，除取得您授权或法律法规另有规定外，我们会将该类个人信息做匿名化、去标识化处理。

(3)如您不希望使用上述功能，您可以随时通过您的设备系统的相关功能设置页面开启/取消蓝牙功能。您开启设备蓝牙功能即视为您授权我们可以访问、获取、收集、使用您的该等个人信息;但当您关闭设备蓝牙功能后，我们将不再收集该信息，也无法再为您提供上述与之对应的服务;但除非您依照法律的规定删除了您的个人信息，否则您的取消行为不会影响我们基于您之前的授权进行的您个人信息的处理、存储。同时，如果您使用iOS设备使用上述功能并且您的设备系统为iOS13.1及以上时，您也可以随时通过使用我们的产品与/或服务的相关功能设置页面开启/取消该权限。iOS端迅康测评APP的 Siri使用权限设置路径为:“我的一设置一隐私权限设置一允许迅康测评访问蓝牙”。当您更新APP版本后，未经您的明确同意，我们不会更改您之前设置的权限状态。

4.3从第三方间接收集的您的个人信息

(1)您理解并知悉，您向外部第三方(迅康测评旗下关联公司不在此限)提供的个人信息，或外部第三方收集的您的个人信息，我们无法获取，更不会使用非常规方式(如:恶意干预对方系列APP数据)擅自以软件程序获得您的个人信息。

迅康测评可能因业务发展的需要而确实需要从第三方间接收集(如共享等)您的个人信息的，且由我们直接或共同为您提供产品或服务的，我们(或第三方)在收集前会向您明示共享的您个人信息的来源、类型、使用目的、方式和所用于的业务功能、授权同意范围(如果使用方式和范围超出您在第三方原授权范围的，我们会再次征得您的授权同意)。我们的某些产品或服务由第三方业务合作伙伴提供或共同提供时，为了必要且合理的开展业务，我们可能会从部分业务合作伙伴处间接收集的您的部分信息、其他方使用我们的产品与/或服务时所提供有关您的信息。

(2)我们的专业安全团队对个人信息将进行安全加固(包括敏感信息报备、敏感信息加密存储、访问权限控制等)。我们会使用不低于我们对自身用户个人信息同等的保护手段与措施对间接获取的个人信息进行保护。

4.4出于其他合理且必要的目的

(1)如前文所述，如果某一需要收集您的个人信息的功能或产品/服务未能在本隐私政策中予以说明的，或者我们超出了与收集您的个人信息时所声称的目的及具有直接或合理关联范围的，我们将在收集和使用您的个人信息前，通过更新本隐私政策、页面提示、弹窗、站内信、网站公告或其他便于您获知的方式另行向您说明，并为您提供自主选择同意的方式，且在征得您明示同意后收集和使用。

(2)您理解并同意，在以下情况下，我们无需取得您的授权同意即可收集和使用您的个人信息:

a)与国家安全、国防安全有关的;

b)与公共安全、公共卫生、重大公共利益有关的;

c)与犯罪侦查、起诉、审判和判决执行等直接相关的;

d)出于维护您或其他个人的生命、财产等重大合法权益但又很难得到您本人同意的;

e)所收集的信息是您自行向社会公开的或者是从合法公开的渠道(如合法的新闻报道、政府信息公开等渠道)中收集到的;

f) 根据与您签订和履行相关协议或其他书面文件所必需的;

g)用于维护我们的产品与/或服务的安全稳定运行所必需的，例如发现、处置产品与/或服务的故障;

h)有权机关的要求、法律法规等规定的其他情形。

Ⅴ、我们如何共享、转让、公开披露您的个人信息

1.共享

我们重视对您的个人信息的保护，您的个人信息是我们为您提供产品与/或服务的重要依据和组成部分，对于您的个人信息，我们仅在本隐私政策所述目的和范围内或根据法律法规的要求收集和使用，并严格保密。

通常情况下，我们不会与任何公司、组织和个人共享您的个人信息，但以下情况除外:

1.1事先已获得您的明确授权或同意;

1.2 根据适用的法律法规、法律程序、政府的强制命令或司法裁定而需共享您的个人信息;

1.3 在法律要求或允许的范围内，为了保护迅康测评及其用户或社会公众的利益、财产或安全免遭损害而有必要提供您的个人信息给第三方;

2.转让

除非获取您的明确同意，我们不会将您的个人信息转让给任何公司、组织或个人。如果发生合并、收购或破产清算，将可能涉及到个人信息转让，此种情况下我们会要求新的持有您个人信息的公司、组织继续受本隐私政策的约束。如果本隐私政策中约定的个人信息的收集、处理方式发生任何改变，该公司、组织将重新向您征求授权同意。

3.公开披露

3.1公开披露是指向社会或不特定人群发布信息的行为。除了因需要对违规账号、欺诈行为等进行处罚公告、公布中奖/获胜者等名单时脱敏展示相关信息等必要事宜而进行的必要披露外，我们不会对您的个人信息进行公开披露，如具备合理事由确需公开披露的，我们会在公开披露前向您告知公开披露的信息的目的、类型(如涉及您的个人敏感信息的，我们还会向您告知涉及的敏感信息的内容)，并在征得您的授权同意后再公开披露，但法律法规另有规定的或本政策另有约定的除外。

3.2 对于公开披露的您的个人信息，我们会在收到公开披露申请后第一时间且审慎审查其正当性、合理性、合法性，并在公开披露时和公开披露后采取最严格个人信息安全保护措施和手段对其进行保护。

4.共享、转让、公开披露您的个人信息的例外

请您知悉，根据法律法规的规定，在下述情况下，共享、转让、公开披露您的个人信息无需事先征得您的授权同意:

4.1

与国家安全、国防安全直接相关的;

4.2

与公共安全、公共卫生、重大公共利益直接相关的;

4.3

与犯罪侦查、起诉、审判和判决执行等直接相关的;或根据法律法规的要求、行政机关或公检法等有权机关的要求的;

4.4

出于维护您或其他个人的生命、财产等重大合法权益但又很难得到您本人同意的;

4.54.5

个人信息是您自行向社会公开的或者是从合法公开的渠道(如合法的新闻报道、政府信息公开等渠道)中收集到的;

4.6

根据与您签订和履行相关协议或其他书面文件所必需的;

4.7

法律法规等规定的其他情形。

Ⅵ、关于使用Cookie和同类技术

1.在您使用我们的产品与/或服务时，我们可能会使用 Cookie和同类技术收集您的一些个人信息，包括:您访问网站的习惯、您的浏览信息、您的登录信息，Cookie和同类技术收集该类信息是为了您使用我们的产品与/或服务的必要、简化您重复操作的步骤(如注册、登录)、便于您查看使用历史(如视频观看历史)、向您提供更切合您个人需要的服务内容和您可能更感兴趣的内容、保护您的信息和账号安全性、提升我们的产品和服务等。

2.如果您拒绝我们使用Cookie及同类技术收集和使用您的相关信息，您可在浏览器具备该功能的前提下，通过您的浏览器的设置以管理、(部分/全部)拒绝 Cookie与/或同类技术;或删除已经储存在您的计算机、移动设备或其他装置内的Cookie与/或同类技术，从而实现我们无法全部或部分追踪您的个人信息。您如需详细了解如何更改浏览器设置，请具体查看您使用的浏览器的相关设置页面。

您理解并知悉:我们的某些产品/服务只能通过使用Cookie或同类技术才可得到实现，如您拒绝使用或删除的，您可能将无法正常使用我们的相关产品与/或服务或无法通过我们的产品与/或服务获得最佳的服务体验，同时也可能会对您的信息保护和账号安全性造成一定的影响。

Ⅶ、我们对您个人信息的存储与保护

1.信息存储

1.1存储地点:我们依照法律法规的规定，仅会将收集到的有关您的个人信息存储于中华人民共和国境内。目前我们暂时不存在跨境存储您的个人信息或向境外提供个人信息的场景。如果确有必要进行数据的跨境传输，我们会单独向您明确告知(包括数据出境的目的、接收方、使用方式与范围、使用内容、安全保障措施、安全风险等)，并征得您的授权同意，我们会确保数据接收方有充足的数据保护能力来保护您的个人信息。

1.2存储期限:我们在为您提供满足产品和服务目的所必需且最短的期间内保留您的个人信息，例如:当您使用我们的注册及会员功能时，我们需要收集您的手机号码，且在您提供后并在您使用该功能期间，我们需要持续保存您的手机号码，以向您正常提供该功能、保障您的账号和系统安全。此外，我们或对您的相关信息保存至相关法律所规定的必要期限(例如《电子商务法》规定:商品和服务信息、交易信息保存时间自交易完成之日起不少于三年)。

1.3在超出上述存储期限后，我们会对您的个人信息进行删除或匿名化处理。

2.信息保护

2.1您的个人信息安全对于我们至关重要。我们将严格遵守相关法律法规，采取业内认可的合理可行的措施，保护您的个人信息。防止信息遭到未经授权的访问、披露、使用、修改，避免信息损坏或丢失。

2.2我们使用符合业界标准的安全防护措施保护您提供的个人信息，防止数据遭到未经授权的访问、公开披露、使用、修改，防止数据发生损坏或丢失。我们会采取一切合理可行的措施，保护您的个人信息。例如使用SSL对数据进行加密保护;我们已建立访问控制机制，确保只有授权人员才可以访问个人信息;我们不时的举办安全和隐私保护培训课程，加强员工对于保护个人信息重要性的认识。

2.3 为防止安全事故的发生，我们成立了专门的数据安全部门并制定了妥善的预警机制和应急预案。一旦发生个人信息安全事件，我们将按照法律法规的要求，及时向您告知:安全事件的基本情况和可能的影响、我们已采取或将要采取的处置措施、您可自主防范和降低风险的建议和对您的补救措施，并立即启动应急预案，力求将损失最小化。我们将及时将事件相关情况以电话、推送通知等方式告知您。

Ⅷ、您管理个人信息的权利

我们理解您对个人信息的关注，并尽全力确保您对于自己个人信息访问、更正、删除以及撤回授权的权利，以使您拥有充分的能力保障您的隐私和安全。您的权利包括:

1.您有权访问您的个人信息

您可以按照我们提供的产品和服务的相关说明(或设置)，对您已提供给我们的相关个人信息进行查阅。包括:

1.1 账号信息:您可以通过相关产品页面随时登陆您的个人中心，以访问您账号中的个人资料信息，包括:头像、昵称、UID、二维码名片、性别、出生年月、个人签名等;

1.2 使用信息:您可以通过相关产品页面随时查阅您的使用信息，包括:动态发布内容、投稿内容、收藏记录、历史记录、订单信息、地址信息、账单记录等;

1.3 其他信息:如您在访问过程中遇到操作问题需获取其他前述无法获知的个人信息内容，您可通过本隐私政策提供的方式联系我们。

2.您有权更正/修改您的个人信息

当您发现您提供给我们的个人信息存在登记错误、不完整或有更新的，您可在我们的产品和/或服务中更正/修改您的个人信息。

2.1对于您的部分个人信息，我们在产品的相关功能页面为您提供了操作设置，您可以直接进行更正/修改，例如:“头像/昵称/性别/出生年月/个人签名”在迅康测评iOS客户端中的更正/修改路径为:点击头像-编辑资料。

2.2对于您在行使上述权利过程中遇到的困难，或者其他可能目前无法向您提供在线自行更正/修改服务的，经过对您身份的验证，且更正/修改不影响信息的客观性和准确性的情况下，您有权对错误或不完整的信息作出更正或修改，或在特定情况下，尤其是数据错误时，通过我们公布的反馈与报错等措施将您的更正/修改申请提交给我们，要求我们更正或修改您的数据，但法律法规另有规定的除外。但出于安全性和身份识别的考虑，您可能无法修改注册时提交的某些初始注册信息。

3.您有权删除您的个人信息

3.1对于您的部分个人信息，您也可以自行通过我们提供的相关产品和服务的功能页面，主动删除您提供信息。例如:“历史记录”信息在迅康测评iOS客户端中的删除路径为:我的-历史记录-编辑。您也可以自主删除您发布的视频、动态、图片等。一旦您删除后，我们即会对此类信息进行删除或匿名化处理，除非法律法规另有规定。

3.2 当发生以下情况时，您可以直接要求我们删除您的个人信息，但已做匿名化处理或法律法规另有规定的除外:

(1)当我们处理个人信息的行为违反法律法规时;(2) 当我们收集、使用您的个人信息，却未征得您的同意时;

(3) 当我们处理个人信息的行为违反了与您的约定时;

(4)当您注销了迅康测评账号时;(5) 当我们终止服务及运营时。

4.您有权撤回您对个人信息的授权

如前文所述，我们提供的产品和服务的部分功能需要获得您使用设备的相关权限(包括:位置、相机、麦克风、日程安排等，具体以本隐私政策第四条为

准)。您可以在授权后随时撤回(或停止)对该权限的继续授权。当您更新App版本后，未经您的明确同意，我们不会更改您之前设置的权限状态。例如您可以通过移动设备中的“设置-隐私-照片”来关闭您对手机相册的授权。您也可以通过注销账号的方式，永久撤回我们继续收集您个人信息的全部授权。您需理解，当您撤回授权后，我们无法继续为您提供撤回授权所对应的特定功能和/或服务。但您撤回授权的决定，不会影响此前基于您的授权而开展的个人信息处理。

5.您有权注销您的账号

您可以通过在线申请注销或客服或通过其他我们公示的方式申请注销您的账号。

当您注销账号后，您将无法再以该账号登录和使用我们的产品与服务;且该账号在迅康测评及旗下的其他产品与服务使用期间已产生的但未消耗完毕的权益及未来的逾期利益等全部权益将被清除;该账号下的内容、信息、数据、记录等将会被删除或匿名化处理(但法律法规另有规定或监管部门另有要求的除外，如依据《中华人民共和国网络安全法》规定，您的网络操作日志将至少保留六个月的时间);迅康测评账号注销完成后，将无法恢复。

如您在谨慎考虑后仍执意决定注销您的迅康测评账号的，您可以在我们提供的联系方式中联系我们进行注销

我们会在收到您的注销申请，并在验证您的用户身份后的15个工作日内尽快向您回复。如果您在处置您的个人信息时有任何疑问，您可以通过本隐私政策第十一条“联系我们”中公示的联系方式与我们沟通解决。

Ⅸ、我们对未成年人的保护

1.未成年人使用我们的产品与/或服务前应取得其监护人的同意。

如您为未成年人，在使用我们的产品与/或服务前，应在监护人监护、指导下共同阅读本隐私政策且应在监护人明确同意和指导下使用我们的产品与/或服务、提交个人信息。

我们根据国家相关法律的规定保护未成年人的个人信息，只会在法律法规允许、监护人明确同意或保护您的权益所必要的情况下收集、使用或公开披露未成年人的个人信息。

2.若您是未成年人的监护人，当您对您所监护的未成年人的个人信息有相关疑问时，您可以通过本隐私政策第十一条“联系我们”中公示的联系方式与我们沟通解决。如果我们发现在未事先获得可证实的监护人同意的情况下收集了未成年人的个人信息，则会尽快删除相关数据。Ⅹ、我们对去世用户的个人信息保护

1.迅康测评将从2021年11月1日起，根据《个人信息保护法》的相关规定升级死者个人信息保护制度。迅康测评用户(仅限自然人)去世后，其近亲属为了自身的合法、正当利益，可以通过本隐私政策第十一条“联系我们”中公示的联系方式，对去世用户的相关个人信息行使查阅、复制、更正、删除等权利，但是去世用户生前另有安排的除外。

2.您理解并确认，为了充分保护去世用户的个人信息权益，申请行使本条权利的去世用户近亲属需要根据迅康测评的指定流程或客服提示，提交去世用户的身份证明文件、死亡证明文件、申请人的身份证明文件、申请人与去世用户的亲属关系证明文件，并提供申请行使的权利种类、目的。

- **Univariate ordered logistic regression analysis for FAST scores as categorical independent variables**

| **Variables** | **B** | **SE** | **P** | **OR (95 % CI)** |
| --- | --- | --- | --- | --- |
| **no impairment** | 0.82 | 1.22 | 0.50 | 2.28(0.21-25.06) |
| **mild impairment** | 0.87 | 1.23 | 0.48 | 2.39(0.22-26.35) |
| **Moderate impairment** | 0.41 | 1.23 | 0.74 | 1.51(0.14-16.63) |

* The reference values for the categorical independent variables was severe impairment

- **Distribution patterns for each mood state group in the 14-day assessment**

| **Variables** | **Complete 42 records(n=4)** | **Complete ≥31 records(n=48)** | **Complete <31 records(n=48)** |
| --- | --- | --- | --- |
| **(Hypo)manic episode** | 1 | 3 | 9 |
| **Euthymic state** | 3 | 28 | 23 |
| **Depressive episode** | 0 | 13 | 16 |

- **Average daily completion rate for each mood state over 14-day period**

| **Variables** | **day1** | **day2** | **day3** | **day4** | **day5** | **day6** | **day7** | **day8** | **day9** | **day10** | **day11** | **day12** | **day13** | **day14** |
| --- | --- | --- | --- | --- | --- | --- | --- | --- | --- | --- | --- | --- | --- | --- |
| **(Hypo)manic episode** | 2.29 | 2.65 | 2.29 | 2.18 | 2.24 | 2.29 | 2.06 | 1.94 | 2.06 | 1.94 | 2.00 | 2.12 | 2.00 | 1.65 |
| **Euthymic state** | 2.26 | 2.33 | 2.19 | 2.13 | 1.94 | 1.94 | 1.96 | 1.85 | 2.04 | 1.91 | 1.81 | 1.67 | 1.50 | 1.57 |
| **Depressive episode** | 2.17 | 1.97 | 2.10 | 1.62 | 1.76 | 1.79 | 1.79 | 1.62 | 1.62 | 1.55 | 1.79 | 1.52 | 1.55 | 1.21 |
