# Supplementary material for: Demographic and Clinical Characteristics Influencing Ecological Momentary Assessment Compliance in Individuals With Bipolar Disorder: Observational Study
Source: JMIR Form Res. 2026 Jan 7;10:e74223. doi: 10.2196/74223 (PMC12824568; doi:10.2196/74223)
Supplement: Multimedia Appendix 2 [file formative_v10i1e74223_app2.docx]

**Daily Self-assessment Questionnaire**

**Morning assessment**

1.What time did you lie down last night?(e.g., 20:00)

2.What time did you try to fall asleep last night? (e.g., 23:55)

3.How long did it take you to fall asleep? (e.g., 5, unit: minutes)

4.How many times did you wake up last night? (e.g., 2, unit: times)

5.What was the total time spent awake in the middle of the night?(e.g., 10, unit: minutes)

6.What time did you wake up this morning?

7.What time did you get out of bed this morning?

8.How would you rate the quality of your sleep last night? (1=Very poor; 2=Poor; 3=Average; 4=Good; 5=Very good)

9.Did you take any sleep aids last night? (1=Yes; 2=No)

**Random self-assessment**

1.Please describe your current mood? (Very low - Low - Sometimes low - Normal - Sometimes high - High - Very high) (-3 points - 3 points)

2.Please describe your current energy level? (Very tired - Tired - Slightly tired - Normal - Slightly energetic - Energetic - Very energetic) (-3 points - 3 points)

3.Please describe your current activity level? (Very low activity - Low activity - Slightly low activity - Normal - Slightly high activity - High activity - Very high activity) (-3 points - 3 points)

4.Please describe your current anxiety level? (None at all - Slight anxiety - Moderate anxiety - Severe anxiety) (0-3 points)

5.Do you currently experience anger, excitement, or arguments over minor issues? (None at all - Slight - Moderate - Severe) (0-3 points)

6.Please describe your current speech speed? (Very slow - Slow - Slightly slow - Normal - Slightly fast - Fast - Very fast) (-3 points - 3 points)

7.Please describe your current thinking speed? (Very slow - Slow - Slightly slow - Normal - Slightly fast - Fast - Very fast) (-3 points - 3 points)

**Bedtime self-assessment**

1.Today, did anything positive or negative happen in your life? (For example: receiving praise from others; having disagreements or conflicts with others, etc.) (1=Yes (please briefly describe the most important event) ______ 2=No)

1.1 Please assess your feelings or experiences regarding this event.(-3=Very negative/Negative; 0=Neutral; 3=Very positive/Positive)

1.2 Please assess the stress this event has caused you.(-3=No stress at all~3=Extreme stress)

2.Did you take your medication as prescribed today? (1. Yes 2. No (record changes) __________)

3.Did you take a nap or rest during the day? (1=Yes 2=No)

3.1When did you take a nap?

3.2 how long was the nap/rest?

4.Did you smoke today? (1=Yes 2=No)

4.1 How many cigarettes did you smoke?

5.Did you drink alcohol today? (1=Yes 2=No)

5.1 What type of alcohol did you drink?

5.2 How much did you drink (in ml)?

6. Did you engage in heavy physical activity for at least 10 minutes today (activities that require you to exert a lot of effort and breathe more heavily than usual), such as lifting heavy objects, running, swimming, playing soccer, basketball, jumping rope, or participating in aerobics in the gym? (1=Yes 2=No)

6.1 If the answer is 1, how long did you engage in the activity? (e.g., 60, unit: minutes, whole number)

7. Did you engage in moderate-intensity physical activity for at least 10 minutes today (activities that require you to exert moderate effort and breathe slightly more heavily than usual), such as playing Tai Chi, table tennis, or social dancing (excluding walking)? (1=Yes 2=No)

7.1 If the answer is 1, how long did you engage in the activity? (e.g., 100, unit: minutes, whole number)

8. Did you walk for at least 10 minutes today? (1=Yes 2=No)

8.1 If the answer is 1, then ask, how long did you walk? (e.g., 30, unit: minutes, whole number)

9. How long did you spend sitting still today? This includes time spent in a hospital room or other places, sitting at a desk, on a bed, sitting or lying down watching TV, reading, etc. (e.g., 3.5, unit: hours, can take 1 decimal place)
